# The relationship between stigma and help-seeking intentions in college students

**DOI:** 10.1192/j.eurpsy.2021.1052

**Published:** 2021-08-13

**Authors:** B. Abassi, F. Fekih-Romdhane, H. Maktouf, I. Moalla

**Affiliations:** Psychiatry E, Razi Hospital, La Manouba, Tunisia

**Keywords:** mental health, Stigma, Reported and Intended Behavior Scale, help-seeking intentions

## Abstract

**Introduction:**

Few people seek mental health care despite the high prevalence of mental illnesses and their serious consequences. Barriers explaining this lack or absence of help-seeking have been the subject of several studies with stigma being the most prominent barrier.

**Objectives:**

We aimed to assess the relationship between stigma and help-seeking intentions in a sample of Tunisian college students.

**Methods:**

This was a cross-sectional survey. The “Mental Health Knowledge Schedule”, and the “Reported and Intended Behavior Scale” were administered to a total of 714 college students (62.2% female; mean age =20.9).

**Results:**

More than one-third of students (39.6%) felt that it would be “unlikely” or “very unlikely” to seek the help of a health professional for mental health problems. Pearson product moment correlations indicated that help-seeking intentions significantly and positively correlated with behavior (p<0.001, r=0,103) and knowledge (p<0.001, r=0,163). The multiple hierarchical linear regression analysis found that after controlling for demographic variables (gender, age) and other personal-related variables (marital status, family income, living environment, substance use, personal psychiatric history), help-seeking intentions were significant contributors to behaviors and knowledge.

**Conclusions:**

Given that stigma and discrimination against people with mental illness is a real and perceived barrier to seeking help and treatment, psycho-educational campaigns should be put in place to address the negative perceptions and attitudes of the general population about mental health issues, and should be tailored to the needs of specific groups (including students in different disciplines), taking into account their attitudes, fear and concerns about mental illness.

